# Modelling background air pollution exposure in urban environments: Implications for epidemiological research

**DOI:** 10.1016/j.envsoft.2018.02.011

**Published:** 2018-08

**Authors:** Álvaro Gómez-Losada, José Carlos M. Pires, Rafael Pino-Mejías

**Affiliations:** a European Commission, Joint Research Centre (JRC), Edificio Expo. C/ Inca Garcilaso 3, 41092, Seville, Spain; b LEPABE, Departamento de Engenharia Química, Faculdade de Engenharia, Universidade do Porto, Rua Dr. Roberto Frias s/n, 4200-465, Porto, Portugal; c Department of Statistics and Operational Research, University of Seville, Avda. Reina Mercedes s/n, Seville, Spain

**Keywords:** Clustering techniques, Background pollution, Air quality, Time-series analysis, Exposure, Health risk

## Abstract

Background pollution represents the lowest levels of ambient air pollution to which the population is chronically exposed, but few studies have focused on thoroughly characterizing this regime. This study uses clustering statistical techniques as a modelling approach to characterize this pollution regime while deriving reliable information to be used as estimates of exposure in epidemiological studies. The background levels of four key pollutants in five urban areas of Andalusia (Spain) were characterized over an 11-year period (2005–2015) using four widely-known clustering methods. For each pollutant data set, the first (lowest) cluster representative of the background regime was studied using finite mixture models, agglomerative hierarchical clustering, hidden Markov models (*hmm*) and k-means. Clustering method *hmm* outperforms the rest of the techniques used, providing important estimates of exposures related to background pollution as its mean, acuteness and time incidence values in the ambient air for all the air pollutants and sites studied.

## Highlights

• Four clustering techniques were compared to model background air pollution.
• *k-means* and *agglomerative* clustering were approximated to model-based clustering.
• *Hidden Markov models* (*hmm*) outperform the rest of the techniques used.
• Information derived from *hmm* results of interest for epidemiological research.

## 1 Introduction

Determining the population's health risks due to ambient air pollution is critical to the development of effective risk management policies and strategies (Samet and Krewski, 2007). To better understand the adverse health effects associated with air pollution, accurate exposure assessment is essential. Epidemiological studies have provided a substantial body of evidence linking daily concentrations of outdoor air pollution to adverse effects on a range of health outcomes. Studies have tended to focus on the mass concentrations of particles and selected gaseous pollutants, but more insight is required regarding the most harmful sources and components of the air pollution mixture to inform focused public health protection policies (Atkinson et al., 2016).

Background concentration is the ambient level of pollution that is not affected by local sources of pollution (WHO, 1980, Menichini et al., 2007). There are two motivations for focusing on this regime: (i) to better understand the contribution of local sources to total pollutant concentrations; and (ii) to allow the assessment of new pollutant sources that are introduced into the area of study and their impact on local air quality. However, up until now research has not significantly addressed this lowest fraction of pollution as representative of a permanent concentration of ambient air pollution to which the population is chronically exposed. This work focuses on this specific fraction of pollution.

Han et al. (2015) classify the methods to determine the background pollution using four categories: (i) physical methods to identify the regional and local pollution processes via atmospheric variables; (ii) chemical methods to identify the chemical composition of air pollutants; (iii) numerical simulations methods using trajectory models; and (iv) statistical methods. Regarding the latter, Langford et al. (2009) used principal component analysis to describe the local background O_3_ concentrations recorded during 76 days in 30 monitoring sites in Texas. Tchepel et al. (2010) study the contributions to background pollution of PM_10_ from different sources in four monitoring sites in Lisbon (Portugal) during two days, through air quality time series via spectral analysis. Other authors have used clustering techniques to characterize regimes in air pollution. Austin et al. (2012) classify air pollution daily data during six years performing k-means (*km*) and hierarchical clustering for identifying profiles in them. Beaver and Palazoglu (2006) used an aggregated solution of *km* to characterize classes of ozone episodes occurring in the San Francisco Bay. Considerable effort has been made to characterize profiles of key air pollutants (Carslaw and Ropkins, 2012, Carlsaw and Beevers, 2013) since the threshold values that can be considered safe for human health is still under debate. Pioneering research work explored this relationship for O_3_ and PM_10_ (Koop and Tole, 2006), and for PM_2.5_ (Kiesewetter et al., 2015). Background profiles of CO and NO_x_ were studied by Venegas and Mazzeo (2006) in the city of Buenos Aires, and for NO_x_, NO_2_ and O_3_ in the California South Coast Air Basin by Pournazery et al. (2014).

This study proposes the use of statistical clustering techniques as a methodology for the estimation of background pollution in urban environments. To that end, four well-known clustering methods were compared using data obtained from monitoring sites, namely: finite mixture models (*fmm*), agglomerative hierarchical clustering (*hc*), hidden Markov models (*hmm*) and *km*.

This study aims to: (i) evaluate the best clustering statistical method to estimate the background pollution; and (ii) provide model-derived exposure estimates from the best method as inputs for epidemiological research. The best clustering method was assessed according to its ability to cluster the lowest concentrations of ambient air pollution in a consistent manner. To that end, data sets from key pollutants CO, NO_2_, O_3_ and PM_10_ from five monitoring sites in Andalusia (south of Spain) were studied over 11 years.

## 2 Data and methods

### 2.1 Air pollution data

Air quality data (hourly average concentrations of CO, NO_2_, O_3_ and PM_10_) were collected from 2005 to 2015 as independent yearly series for each pollutant. These data were obtained at five monitoring sites exhibiting different typology (suburban, urban) and predominant emission sources (background, traffic). Since monitored data were available on an average hourly basis, daily mean concentrations were calculated when at least 80% of the data were available. A total of 200 yearly data sets, each one consisting of daily average values for a single pollutant and complete years were studied, resulting from 40, 55, 50 and 55 data sets corresponding to the air pollutants CO, NO_2_, O_3_ and PM_10_, respectively (Table 1). In order to favour the heterogeneity both of data and range of pollutant concentrations to study, monitoring sites were selected in three different cities of Andalusia (Córdoba, Jaén and Seville) with different meteorological conditions governing the local air pollutant behaviour. The standard monitoring methods established in European Directive 2008/50/EC (Directive, 2008) were used for air pollutants CO, NO_2_ and O_3_, and beta attenuation monitoring was applied for PM_10_. Air quality monitoring networks are subject to an intense maintenance program to ensure accurate values. Prior to undergoing analysis, the data obtained were validated by the Regional Ministry of Environment and Land Planning of Andalusia.

**Table 1:** Analysed pollutants, classification of monitoring sites and period of study where data were obtained: S-Suburban, U-Urban, B-Background, T-Traffic. Locations are given in X,Y ETRS89-UTM coordinates, zone 30.

City	Site	Location		Type	Main pollution source	Pollutant	Annual periods	Number of data sets
		X	Y					
						CO	2005–07	9
							2010–15	
Córdoba	Asomadilla	343546	4196517	U	B	NO_2_		11
						O_3_	2005–15	11
						PM_10_		11

						CO	2007–15	9
Jaén	Bailén	431261	4216416	U	B	NO_2_	2005–15	11
						O_3_	2010–15	6
						PM_10_	2005–15	11

						NO_2_		11
	Aljarafe	230473	4137017	S	B	O_3_		11
						PM_10_		11

						CO		11
Seville	Bermejales	236063	4137554	S	B	NO_2_		11
						O_3_	2005–15	11
						PM_10_		11

						CO		11
	Torneo	234151	4142873	U	T	NO_2_		11
						O_3_		11
						PM_10_		+11
								
								200

### 2.2 Background pollution estimation

For each independent yearly data set with measurements of a single pollutant a clustering technique was applied. For a clustering result, each cluster represents ranges of concentration values (profiles or regimes of pollution) for a given pollutant that can be associated to an emission source of pollution. This view is based on the Lenschow approach (Lenschow et al., 2001) that assumes that the air pollutant concentrations at a monitoring site correspond to the sum of regional, urban background and local nature contributions. This approach has been used as a prior analysis in source apportionment studies (Belis et al., 2013), and may be applied to urban areas with negligible impact from industrial emissions, as in case of Córdoba, Seville and Jaén.

The concentration measured at a traffic site corresponds to the sum of local traffic, urban and regional background contributions. With regard to an urban or suburban background site, the contributions that explain the ambient pollution correspond to those from the background levels of the city or metropolitan area, respectively, and those of the regional background.

Being a univariate clustering process, the resulting clusters represent certain categorization of the original variables into a set of ranges determined by each cluster. When sorting the cluster according to the associated ranges, the first cluster contains the lowest values of the pollutant and it represents the range of minimum concentrations obtained at a monitoring site. This work focuses specifically on this first cluster, which might represent the magnitude of a kind of chronic exposure concentration experienced by the population along the year. One of the most important advantages of this approach is that it allows the estimation of the first cluster representing the background pollution at any monitoring site and for any air pollutant. The estimation is affected neither by the main type of pollution source present nor by the classification of the monitoring site according to its location. The ability of four clustering techniques to detect the lowest cluster on different air pollutants was compared.

### 2.3 Clustering techniques

*fmm*, *hc*, *hmm* and *km* were used to cluster data obtained from monitoring sites. The aim was to study their ability to detect more than one cluster in data, and therefore to be able to associate the lowest one to the background pollution regime. Because clustering via *fmm* represents the foundational model upon which the rest of the clustering techniques are based, it is explained next. In the interest of space, a description of *hc*, *hmm* and *km* is given in Supplementary Material (SM.) 1.

**Fig. 1:**
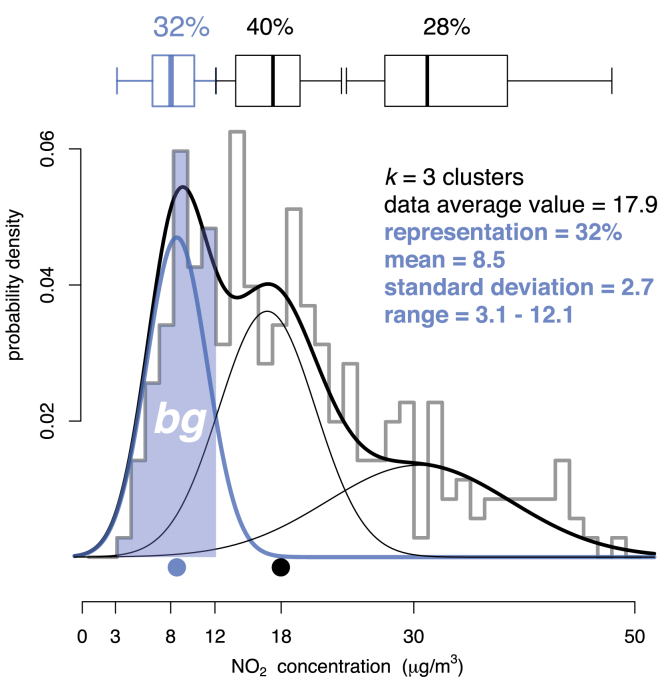
Complete NO_2_ background pollution analysis using finite mixture models in Aljarafe site (Seville) during 2015 (in μg/m^3^). The estimated density of the mixture (thick black line) is obtained after a weighted sum of the three components (*k* = 3 clusters) and superimposed to the histogram of data (grey line). The information regarding the background regime appears in blue (from top to bottom: mixing proportion or representation of background data -32%-, distribution as a box-whisker diagram, shadowed area showing the background -*bg*- data into the histogram, Gaussian curve fitting the data, and the mean value as a blue circle). The rest of information belonging to the second and third regimes is not studied in this work. Each cluster has a different variance. (For interpretation of the references to colour in this figure legend, the reader is referred to the Web version of this article.)

Fig. 1 illustrates the use of *fmm* to model the first cluster, equivalent in this work to the background pollution, in the NO_2_ data distribution (histogram) from the Aljarafe site during 2015 (the information regarding the background pollution appearing in blue). *fmm* represents a model-based strategy for clustering by assuming that each cluster of data is described by a different probability distribution (component). These clusters are combined according to the mixing proportions (representations or weights) that make up the mixture, making the modelling of any multi-modal data set possible because of *fmm's* extreme flexibility (McLachlan and Peel, 2000). In this work, all the distributions were considered univariate Gaussians. The dispersion of the components defining each cluster is given by the standard deviation of the Gaussian distributions. These standard deviations can be constrained as constant across the clusters of the mixture (“E” configuration, equal variances) or allowed to vary between them (“V” configuration, variable variances). Once the number of clusters has been fixed in advance to model the data, the mixing proportion or representation (%), the mean (*m*) and standard deviation (*sd*) of each component parameterizes *fmm*.

Estimating the parameters defining a *fmm* that are most likely to have generated a given data set is referred to as the maximum likelihood estimation (MLE) problem.

Although there are many methods that can be used to estimate the parameters of a *fmm*, the expectation-maximization (EM) algorithm (Dempster et al., 1977) is the most widely used (McLachlan and Krishnan, 2008). The EM algorithm computes the maximum log-likelihood estimates of the mixture iteratively, alternating between two steps, E (the expectation step) and M (the maximisation step), until a convergence criterion is met. The E-step calculates the log-likelihood given the observed data and the current parameters estimate of the mixture, and the M-step maximizes the expected log-likelihood from the previous E-step, providing a new estimation for each parameter. The convergence criterion may be a permitted number of iteration of the algorithm, an acceptable minimum difference (ε) between the parameter estimates at each iteration or both.

Once the EM algorithm converges and the parameters of the *fmm* estimated, the log-likelihood of the data can be calculated. This allows obtaining the BIC (Bayesian information criterion) value (Schwarz, 1978) of the data modelled with a specific *fmm* and for a given number of clusters. Thus, several *fmm* differing in the number of clusters can be proposed to model a data set and the corresponding BIC values calculated. The *fmm* with larger value of the BIC obtained provides the more suitable number of clusters for the data set studied.

The common features shared between *hc*, *hmm* and *km* to *fmm* have simplified the practical implementation of the techniques in this work, and remarkably, provided all of them a common probabilistic foundation (probabilistic clustering). Henceforth, it was possible to address the determination of the most suitable number of clusters *k* in data using the BIC criterion, and thus a comparison of the number of clusters was obtained with the different techniques.

The application of BIC to determine the number of clusters in *fmm* and *hmm* is well known due to its consistency in mixtures from exponential families (Frühwirth-Schnatter, 2006). However, the number of studies describing the application of BIC to *hc* and *km* in the air pollution field is scarce. In this work, the BIC approach avoids the pre-setting of the number of clusters in *hc* and *km* empirically by the user. For each clustering technique and data set studied, an initial number of clusters were proposed (*k*=1, …,9), and the BIC values calculated for each solution. The more suitable number of clusters was chosen according the maximum BIC value. The optimal solution *k* = 1 was also included to verify that no cluster (absence of air pollution profiles) was detectable in the data sets under analysis.

Other Bayesian model selection criteria are possible. General approaches for model selection are Akaike's information criteria (Akaike, 1974), the Deviance Information Criterion (Spiegelhalter et al., 2002), the Integrated Classification Likelihood (Biernacki et al., 2000), and the Focused Information Criteria (Claeskens and Hjort, 2003). The selection of different criteria remains data-dependent and no one criterion is superior to any other in general cases (Xu and Wunsch, 2009). Unfortunately, there seems to be no simple recommendation to guide the use of these criteria, as there are no general results on these methods' performance that apply to all situations. In the short length TS framework as studied in this work, BIC criterion is a parsimonious solution for determining the number of clusters. Next to model selection criteria, other statistics for goodness-of-fit can be found in Mackay Altman (2004) andTitman and Sharples (2008).

The computational implementation of all the cluster techniques was accomplished using the open-source software *R* (R Development Core Team, 2015). Such implementations are available upon request. To determine the optimal number of clusters in data using *fmm*, and the parameters defining each cluster (representation, mean and standard deviation values), the *Mclust* function from the “mclust” package (Fraley et al., 2012) was used, adopting a “V” configuration and setting the iterations of the algorithm to unlimited. This function adopts a default value for relative convergence of the log-likelihood in the EM algorithm of ε = 10^−5^.

#### 2.3.1 *K*-means

*km* algorithm implicitly assumes that the data in each cluster are spherically distributed around the mean (Venables and Ripley, 2002, Hamerly and Elkan, 2003). Therefore, it is possible to derive the *km* algorithm as a special case from the univariate Gaussian *fmm* used in this work, when the variance of the components adopts the “E” configuration (same variance across the components of the mixture).

Data was analysed with *km* using the *kmeans* function from the “stats” package. For each *k* value, the representation of each cluster with respect to the data set size permitted obtaining its representation (weights) as in *fmm.* The common variance was calculated as the sum of the weighted variance of clusters. To parameterize the *km* clustering solutions from a *fmm* approach, the representation, mean value from clusters and their common variance were provided as parameters to the E-step of the EM algorithm (*estep* function from the “mclust” package, “E” configuration). BIC values were later calculated using the *bic* function from this package.

#### 2.3.2 Hidden Markov models

*hmm* belong to the model-based clustering methods which provide a convenient way of formulating an extension of *fmm* to allow for dependent data (McLachlan and Peel, 2000), and the MLE problem can be solved using the EM algorithm (Bulla and Berzel, 2008). Using this clustering technique, each data point represents the observed value of a time series (TS) at time *t*. As in *fmm*, the data are drawn from two or more distributions with different parameters, forming a mixture which can fit multiple modes in TS. An *hmm* is a doubly stochastic process in which an underlying stochastic process (a set of discrete states) can only be observed through another stochastic process that generates a sequence of observations (TS data). Only the TS observations are visible to the observer. The observations of the TS are dependent on the discrete states, such that the marginal distribution of the data is a mixture distribution (as in *fmm*). The data in *hmm* are dependent rather than indepedent draws from the components of the mixture distribution (Visser, 2011). An *hmm* is characterized by a set of states (equivalents to components in *fmm*), an initial probability distribution for the first state, a transition probability matrix linking successive states, and state-dependent probability distributions responsible of generating the TS data. However, just the information characterizing the first cluster is examined (%, *m* and *sd*, as in *fmm*), according to the aim of this study.

The parameters defining the mixtures in TS data was obtained using the *depmix*, function from the “depmixS4” package (Visser and Speekenbrink, 2010), using a tolerance value for the relative convergence of ε = 10^−5^, unlimited EM algorithm iterations and adopting the “V” configuration. To obtain the BIC values from each clustering solution, an *ad hoc* R function was designed, considering the parameters of the mixtures and the size of the data sets. To check the validity of the modelling results obtained with the “depmixS4” package, the “HiddenMarkov” (Harte, 2015) and “HMM” (Himmelmann, 2010) libraries were also used, and negligible differences were found in the parameter estimates.

#### 2.3.3 Agglomerative hierarchical clustering

To define the proximity between clusters in *hc*, an approach equivalent to *km* was used. Ward's method (Ward, 1963) attempts to minimize the sum of squared distances of data from their cluster means (Clarke et al., 2009, Everitt et al., 2011), providing homogeneous, spherical clusters around the cluster means, an approach that is analogous to *km* when dealing with hierarchical clustering (Tan et al., 2006).

To perform *hc* for the constrained Gaussian model (“E” configuration), the procedure followed by Fraley and Raftery (1998), later implemented in Venables and Ripley (2002), was applied using the function *hc* from the “mclust” package, adapted to univariate data. The parameterization of each candidate cluster solutions was obtained using the EM algorithm (*em* function from the same package, “E” configuration) and the BIC values by using the *bic* function from this package.

## 3 Results and discussion

### 3.1 Background regime study at one site during one year

**Fig. 2:**
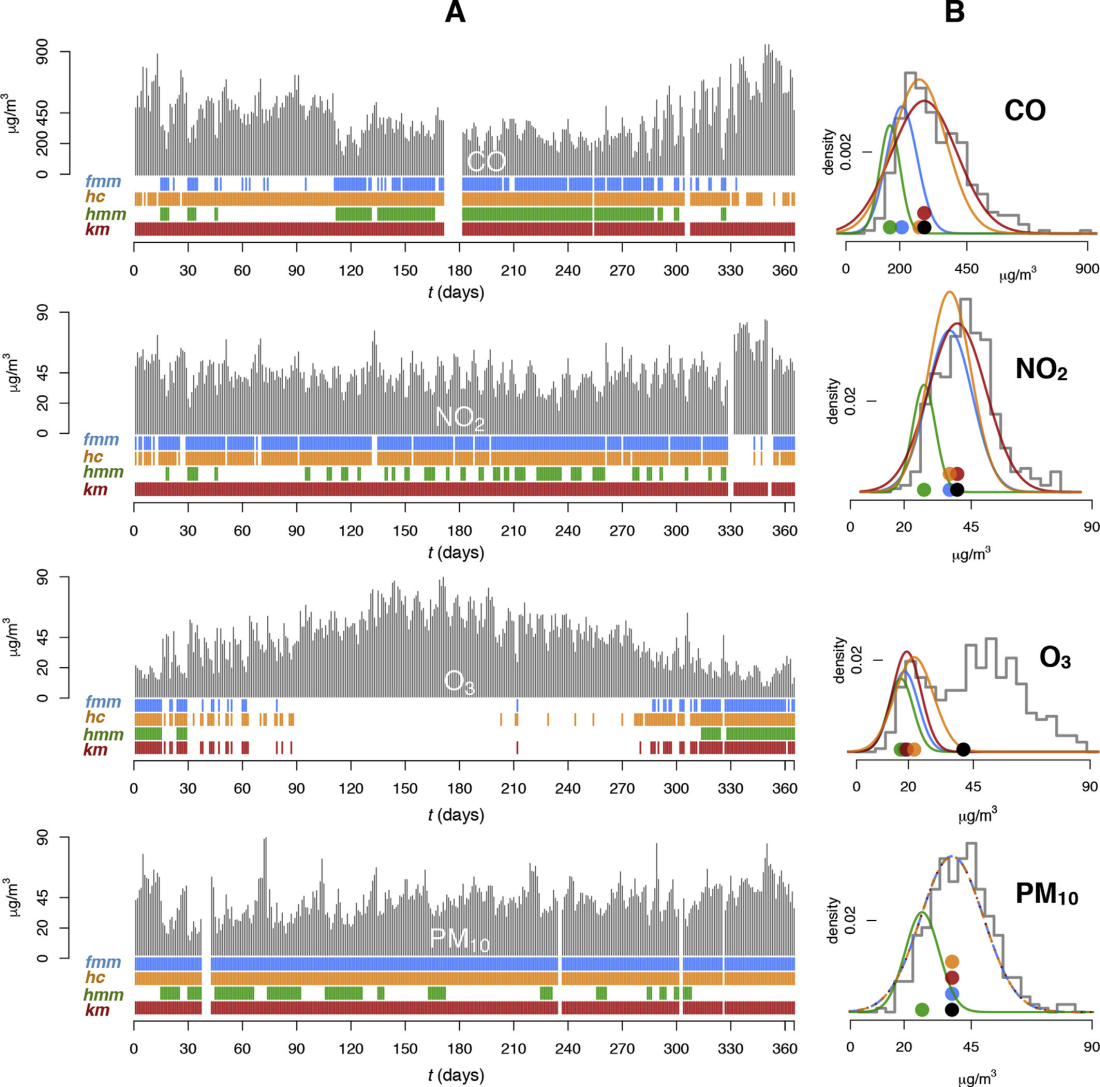
First cluster analyses in Torneo site during 2015 applying the four clustering techniques for CO, NO_2_, O_3_ and PM_10_ data sets. In **A**, pollutant daily concentrations (in μg/m^3^) throughout the year are represented (in grey), and below, each coloured segment corresponds to days grouped in the first cluster of data after applying each technique (*fmm* in blue, *hc* in orange, *hmm* in green, and *km* in red). In **B**, the first cluster is characterized by its Gaussian curve according to the used technique and superimposed to the histogram of data (in grey). Coloured circles represent the average value of the first cluster (average value of the background pollution during the year), and the black circle, the annual average value of the pollutant concentrations. (For interpretation of the references to colour in this figure legend, the reader is referred to the Web version of this article.)

Fig. 2 illustrates the different graphical results corresponding to the first cluster analyses using the four clustering techniques (*fmm*, *hc*, *hmm* and *km*) for the air pollutants studied at the Torneo site during 2015. The corresponding numerical results are given in Table 2. In Fig. 2A, below the daily average concentrations, the different coloured segments indicate days in which no external contributions are detected according to the clustering technique used (daily data grouped into the first cluster). The ability to detect clusters in data is manifested through the *k* value in Table 3. In CO, NO_2_ and PM_10_ pollutants, *km* (red segments) does not detect clusters in data, as *fmm* and *hc* in PM_10_ (blue and orange segments, respectively). Except by *hmm* (green segments), the first cluster detected in NO_2_ is markedly unspecific, since the data grouped in it corresponds to almost the whole range of concentrations during the whole year. Therefore, *hmm* reveals a higher resolution for detecting background concentrations in all the pollutants studied. The four techniques distinguish clusters in O_3_ data, possibly due to the distinct differences in concentration ranges experienced during the year (maximum values in summer and lowest in winter), where any of these clustering solutions would have been potentially valid.

**Table 2:** Analysis of the first cluster (background regime) in Torneo site for 2015 (in μg/m^3^) using different clustering techniques. *k*: number of clusters detected in data; *m* and *sd*: mean and standard deviation of the background regime; *M*: annual concentration average value.

Pollutant	Clustering	*k*	Background concentrations (min-max)	Cluster size. Days (%)	*m*	*sd*	*M*
CO	*fmm*	2	67.8–274.7	183 (52)	207.7	47.9	291.3
	*hc*	2	67.8–499.9	331 (94)	254.2	89.7	
	*hmm*	3	67.8–344.0	183 (52)	199.9	52.7	
	*km*	1	67.8–872.6	351 (100)	291.3	122.6	

NO_2_	*fmm*	2	14.9–51.4	318 (88)	37.5	8.4	39.8
	*hc*	2	14.9–50.5	315 (88)	38.1	8.9	
	*hmm*	3	14.9–34.1	91 (25)	27.8	4.5	
	*km*	1	14.9–74.6	360 (100)	39.8	10.8	

O_3_	*fmm*	2	7.3–26.0	95 (26)	18.5	5.3	41.4
	*hc*	3	7.3–36.2	140 (38)	21.7	7.4	
	*hmm*	4	7.3–28.9	70 (19)	17.1	4.8	
	*km*	4	7.3–28.5	110 (30)	19.3	5.0	

PM_10_	*fmm*	1	9.8–80.4	355 (100)	37.9	11.7	37.9
	*hc*	1	9.8–80.4	355 (100)	37.9	11.7	
	*hmm*	3	9.8–45.2	129 (36)	26.7	6.3	
	*km*	1	9.8–80.4	355 (100)	37.9	11.7

**Table 3:** Proportion of data sets by number of cluster detected (*k*), clustering technique and air pollutant.

Cluster detection	Technique	Counting of data sets/available data sets by pollutants					Total %
		CO	NO_2_	O_3_	PM_10_	Total	
		Number of data sets by pollutant					
		40	55	50	55		
*k* = 1	*fmm*	5/40	18/55	6/50	6/55	35/200	17.5
	*hc*	7/40	14/55	6/50	7/55	34/200	17.0
	*hmm*	1/40	0/55	0/50	1/55	2/200	1.0
	*km*	13/40	31/55	18/50	24/55	86/200	43.0

*k* > 1	*fmm*	35/40	37/55	44/50	49/55	165/200	82.5
	*hc*	33/40	41/55	44/50	48/55	166/200	83.0
	***hmm***	39/40	55/55	50/50	54/55	198/200	**99.0**
	*km*	27/40	24/55	32/50	31/55	114/200	57.0

In bold highest value.

Fig. 2B is equivalent to the cluster analyses represented in Fig. 2A, following the same approach as in Fig. 1, except the density of the mixture is not represented by simplicity. It reveals the Gaussian curves characterizing the first cluster detected by the clustering methods, superimposed to the histogram of data (in grey). Coloured circles represent the average value of the first cluster (*m*), while the black circle represents the annual average value of the concentrations (*M*). As expected, when a technique does not detect clusters in data, the average value of the first (and single) cluster coincides with the average value of the data (PM_10_ clustering in Fig. 1B for *fmm*, *hc* and *km*), and only one Gaussian component models the data.

Table 2 provides more valuable information. Focusing on the first cluster, the Gaussian curves provide the spread of the data (*sd*) around their means (*m*), indicating the strength of the background exposure to the different pollutants. In this analysis, this information can only be consistently obtained in all the pollutants by means of the *hmm* clustering. The time incidence (representation) of the background regime cluster over the whole data set is given by the size of this cluster (%), to measure the proportion of the year in which the population is exposed to the background pollution, characterized by its *m* and *sd* values.

### 3.2 Selection of the best clustering technique

**Fig. 3:**
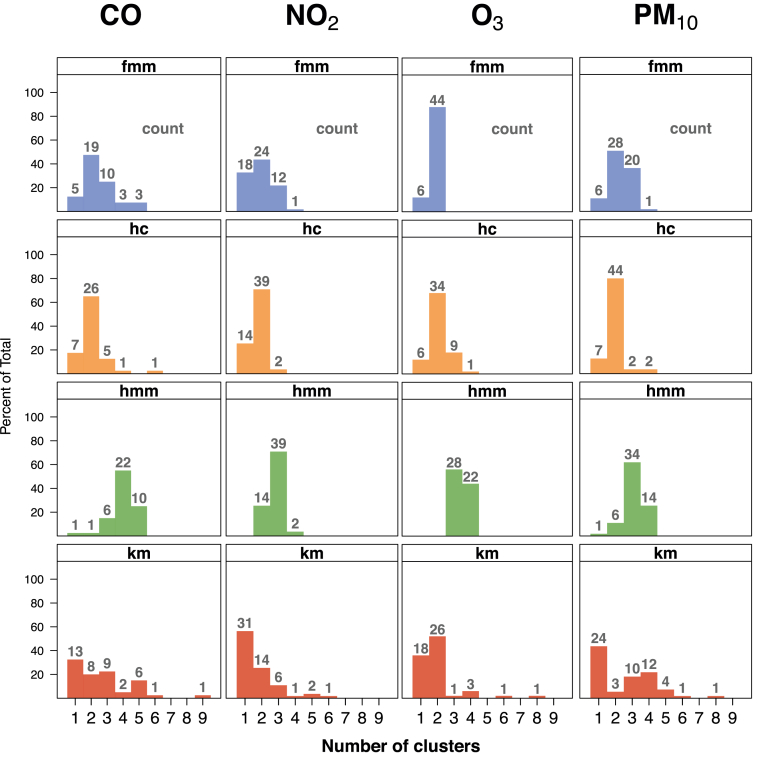
Counting of sets of data (numbers in grey) according to the clusters detected in them, given the pollutants and techniques studied. Same colour code as in Fig. 2. (For interpretation of the references to colour in this figure legend, the reader is referred to the Web version of this article.)

As seen in the previous section, information related to the first cluster of data allows a full description of the background pollution (%, *m* and *sd*). However, this description is possible because it is based on the resolution of clustering techniques that detect more than one cluster in data (*k* > 1). Otherwise, the only detected cluster would parameterize the entire data set with simply one Gaussian curve and would therefore not provide any valuable information. Fig. 3 illustrates clustering methods’ ability to achieve this end, with the number above the bars representing the data sets described with a specific number of clusters for a given technique and pollutant. Table 3 summarizes this information, concluding that *hmm* is the most suitable technique to detect regimes in data and therefore, to describe the background pollution in them (*k* > 1 case, 99%). Meanwhile *km* (57%) is an unsuitable technique, with *fmm* (82.5%) and *hc* (83%) in an intermediate position.

This superior performance of *hmm* may be due to its ability to capture the dynamic behaviour of TS, governed by the Markov property, based on the linkage between subsequent and previous values in the TS, the order of the observations being important. This could suggest that this underlying information contained in TS is not entirely conveyed in *fmm, hc*, and in particular in *km*, or at least, in those cases when these latter techniques detect just a single cluster in data (*k* = 1: *fmm* 17.5%, *hc* 17%, and *km* 43% of the cases). It arises as a natural question if the lower performance of *fmm*, *hc* and *km* is due to their use of dependent data (TS) such as monitored data. However, the literature which applies the referred techniques to TS data (see the review papers and references therein from Aghabozorgi et al., 2015 and Liao, 2005) is vast.

The quality of a clustering solution is difficult to define (Pereira Rodrigues and Gama, 2007). The focus adopted in this work was simply to select the technique with a consistent ability to partition data in more than one cluster, in order to assign the lowest cluster to the background regime. Beyond the scope of this study, a clustering validation based on the comparison of the resulting cluster structures obtained on every data set by the different techniques is currently being considered for further research. In this work *hmm* possesses good clustering properties related to the aim of this work and data sets studied, as long as they fulfil the criteria given by Han et al. (2012): (i) interpretability and usability, (ii) discovery of clusters with arbitrary shape, (iii) ability to deal with noisy data, (iv) scalability (results not shown), and (v) minimum requirement of information provided by the user.

### 3.3 Implications for epidemiological research

**Fig. 4:**
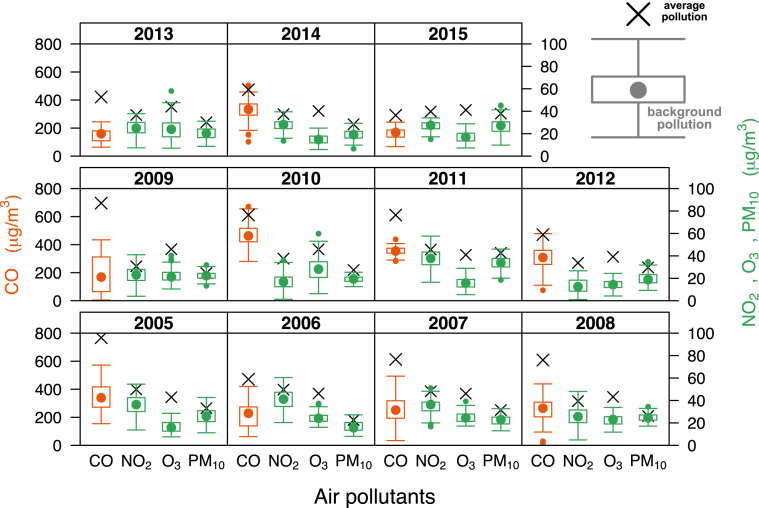
Evolution of the background air pollution of CO, NO_2_, O_3_ and PM_10_ from 2005 to 2011 in Torneo site using *hmm* as clustering technique. The distribution of the background regime is given using box-whisker plots, with the CO concentrations referred to the left axis. The annual average pollution (*M*) of every pollutant is represented by means of a black cross.

The graphical result of the evolution of the background pollution at the Torneo site estimated with *hmm* over 11 years is represented in Fig. 4. The numerical characterization of the background pollution of all sites is given in SM.2, and the graphical representation of the remaining sites in SM. 3. Coinciding with Moreno et al. (2009), background concentrations in cities experienced daily variations indicating that they may be influenced by regional air quality and indirectly by local sources.

As seen in the previous section, *hmm* provides two important features related to background pollution exposure, namely: 1) concentrations, as a quantitative expression of this minimum but permanent abundance of pollution in ambient air, and in this work analysed for CO, NO_2_, O_3_ and PM_10_, and 2) the interval time throughout the year to which this abundance is present. According to the WHO (2013) with respect to PM_10_ pollutant, there is no evidence of a safe level of exposure or a threshold below which no adverse health effects occur. New threshold values and estimates of exposure for this air pollutant, or any other, could now be used in epidemiological studies after applying *hmm* to air pollution. These estimates can be based on the range of concentration of background pollution, their mean (or median) and standard deviation values (as indicative of their acuteness), the quantitative relation between the average pollution to background pollution, or their time incidence (%). Also, background pollution can be studied from a single or multi-pollutant perspective. The background pollution data in this work was estimated on a daily means aggregation basis. However, the scalability of *hmm* allows analyses on hourly data, increasing its resolution.

## 4 Conclusions

Aiming to propose a valid clustering technique to estimate the background pollution in urban environments, four well-known clustering techniques were compared under the same probabilistic framework. The use of *fmm* and *hmm* are widely used to cluster data. However, the approximation of *hc* and *km* to a model-based clustering is scarce in the air pollution literature. These clustering methods were applied on 200 heterogeneous data sets to evaluate their ability to detect background pollution in a consistent manner. *hmm* outperformed with respect to the rest of clustering techniques studied. The information obtained from *hmm* when analysing background pollution may result of interest for epidemiological research in that it provides a full characterization of the background pollution. Mean, standard deviation and representation of background pollution may be used as estimates of exposure to this fraction of pollution in ambient air, and hence to better understand the implications of background pollution on the population's health.

## Data and software availability

The data used in this study were kindly provided by the Regional Ministry of Environment and Land Planning of Andalusia (Seville, Spain). Please contact the corresponding author for any enquiries. Models were implemented using the open-source programming environment R, version 3.2.2 (R Development Core Team, 2015). This software is available for download from www.r-project.org and runs on UNIX, Windows and MacOS platforms. Source codes used in this study are available upon request.

## Disclaimer

The authors declare that they have no actual or potential competing financial interest. The views expressed are purely those of the author and may not in any circumstances be regarded as stating an official position of the European Commission.
