# Supplementary material for: Modelling background air pollution exposure in urban environments: Implications for epidemiological research
Source: Environ Model Softw. 2018 Aug;106:13–21. doi: 10.1016/j.envsoft.2018.02.011 (PMC6018063; doi:10.1016/j.envsoft.2018.02.011)
Supplement: Supplementary data [file mmc1.pdf]

Supplementary material

Modelling background air pollution exposure in urban environments: implications for epidemiological research

Álvaro Gómez-Losada[(1,*)], José Carlos M. Pires[(2)], Rafael Pino-Mejías[(3)]

[(1)] *European Commission, Joint Research Centre (JRC), Edificio Expo. C/ Inca Garcilaso 3, 41092 Seville, Spain.*

[(2)] *LEPABE, Departamento de Engenharia Química, Faculdade de Engenharia, Universidade do Porto. Rua Dr. Roberto Frias s/n, 4200-465 Porto, Portugal.*

[(3)] *Departamento de Estadística e Investigación Operativa, Facultad de Matemáticas, Universidad de Sevilla. C/ Tarfia s/n, 41012 Sevilla, España.*

Contents

- SM.1. Description of *km*, *hmm* and *hc* clustering techniques used in this work.
- SM.2. Characterization of background pollution from studied monitoring sites using *hmm*.
- SM.3. Graphical representation of the background pollution evolution.
- Supplementary Material references.

*Corresponding author: Álvaro Gómez-Losada (e-mail: alvaro.gomez-losada@ec.europa.eu). January 11, 2018

Supplementary material submitted to *Environmental Modelling & Software*.

SM.1

k-means (*km*)

km (Hartigan and Wong, 1979) partitions data into a pre-specified number of k, non-overlapping clusters, assigning each data to one of the clusters, and providing the mean (centres) of each of the k clusters. The algorithm initially assign a different mean value to each cluster of data, and iteratively relocates both data and means into clusters until the total within-cluster variation over each data to its closest mean is as small as possible. This means that data in each cluster are modelled as lying within spheres with the same radius around the cluster centres (Raykov et al., 2016).

Hidden Markov models (*hmm*)

A *hmm* is a doubly stochastic model in which an underlying stochastic process that is not observable (i.e. it is hidden) can only be observed through a sequence of observations. The stochastic process that is not observable is an underlying Markov chain, a series of hidden states (state variables); and the other is the observation sequence (the TS) determined by the current hidden state of the given Markov chain, the outcome of a certain hidden state. Only the TS observations are visible to the observer.

Let $Y_{1:T} := (Y_1, Y_2, \ldots, Y_T)$ be a TS of length T and let $S_{1:T} := (S_1, S_2, \ldots, S_T)$ the *states variables*, these latter being hidden to the observer. These variables S_t are elements from a finite set $\mathcal{S} = \{1, \ldots, k\}$ such that it can be written $S_t = i$, $i \in \mathcal{S}$. Set $\mathcal{S}$ is called the *state-space* of the *hmm*, and k is the number of states of the model. Observations Y_t are dependent on the state variables S_t such that the distribution of Y_t can be written as: $f_i(Y_t) := f(Y_t|S_t = i)$. Because set $\mathcal{S}$ is finite, this means that the marginal distribution of the data (the temporal serie) is a mixture distribution with k components:

$$f\ (Y_t) = \sum_{i=1}^{k} \pi_i\ f_i(Y_t)$$

where π_i are the mixing proportions in which every component (cluster) occurs with the constraints: $\pi_i \geq 0$, for $i \in \{1, \ldots, k\}$, which sum to 1. Each $f_i(\cdot)$ is the conditional distribution of the data in component i and is designated as the *state-dependent distribution* of the model. In this work, $f_i(\cdot)$ is a Gaussian distribution. The figure below shows the dependency graph in an *hmm*:

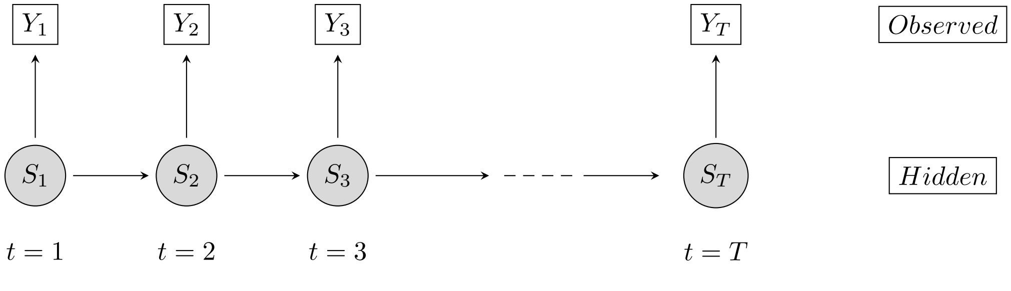

The Markov property in hmms is determined by the dependence between the states, which can be expressed:

$$P(S_t|S_1, \ldots, S_{t-1}) = P(S_t|S_{t-1})$$

and which are referred to as *transition probabilities*. These latter probabilities are denoted by matrix $A(t)$ with entries :

$$a_{ij}(t) = P(S_t = j|S_{t-1} = i) \qquad i, j = 1, \ldots, k$$

and constraints:

$$\sum_{j=1}^{K} a_{ij}(t) = 1, \text{ for each } i; \ \ a_{ij} \geq 0$$

The fundamental assumption of a dependent mixture model is that at any point in time, the observations are distributed as a mixture with k components (clusters or states), and that time-dependencies between the observations are due to time-dependencies between the mixture components (i.e. transition probabilities between the components) (Visser and Speekenbrik, 2010).

An illustration of an *hmm* with 2-states (k=2) is given in Figure SM.1. In this figure, the transitions of one hidden state to another state generating the observations of an annual TS ($T = 365$) are first depicted and then the elements of an *hmm* are defined. For the sake of simplicity, this example uses a two-state *hmm* and the first five observations (from the first day -$t = 1$- to the fifth -$t = 5$-) of the TS are explained. Hidden states are denoted by circles and possible transitions among hidden states by arrows, with their probabilities given. The path generating the observation is indicated by highlighted blue arrows and gray circles. In the beginning ($t = 1$), the Markov chain is initialized according to the initial state probability distribution $\boldsymbol{\delta} = (1, 0)$ and starts at state 1. Then the hidden state transfers from the initial state to the next state according to a transition probability matrix ($\mathbf{A}$), which describes the probabilities for all the transitions. As elements of this matrix are probabilities, they are non-negatives, no greater than 1 and each of the rows sum to unity.

Each of the hidden states addresses an associated statistical distribution from which the data are generated. These distributions are referred to in the literature as emission probabilities denoted by B. In this work, this distribution is represented by a weighted sum of Gaussian densities. In Figure SM.1, they are represented by two Gaussian densities, $N(15, 5)$ and $N(35, 5)$, contributing equally ($\pi_1 = \pi_2 = 0.5$) to fitting the shape (histogram) of the TS. The example finishes at time $t = 5$ after having generated five observations of the TS. Lower concentration values in the TS are generated by the Gaussian distribution associated with state 1, while higher ones are generated by the Gaussian distribution associated with state 2. This observation leads to the formation of two groups of the TS observations depending on their concentration values. This idea helps to clarify the role of hidden states as elements that cluster the TS observations, modeling the temporal heterogeneity of any given TS.

Next, the elements defining an *hmm* may be given:

1. The number of states of the model, k. The individual states are denoted as $\mathcal{S} = \{S_1, \ldots, S_k\}$ and the state at time t is denoted by q_t.
2. The initial state probability distribution, determining in which state the Markov chain starts to transition at $t = 1$, defined as $\boldsymbol{\delta_i} = P(S_1 = i)$, $i = 1, \ldots, k$.
3. The state transition probability matrix $\mathbf{A}$=$\{a_{ij}\}$, with elements:

   $$a_{ij} = P(S_t = j | S_{t-1} = i) \qquad i, j = 1, \ldots, k$$

   indicating the probability the state at time t (j) given the state at time $t - 1$ (i).
4. M, the number of distinct observations of the TS for each state. The individual observations are denoted by $V = \{v_1, v_2, \ldots, v_M\}$.
5. The emission probability distribution in state S_i, $B = \{b_i(k)\}$, where $b_i(k) = P(v_k | q_t = S_i)$, is the probability that a particular observation of the TS is emitted in a state S_i at time t, $i = 1, \ldots, k$, $k = 1, \ldots, M$. This element of the *hmm* includes the parameters of the weighted sum of k Gaussian distributions: the weighting coefficient (π_i), the mean (m_i) and the standard deviation (sd_i) values, $i = 1, \ldots, k$, of the Gaussian distributions. The weighting coefficients satisfy the constraint that their values sum to unity.

A comprehensive account of the *hmms* can be found at Ibe (2013), Visser et al. (2009, 2010, 2011) and Zucchini and MacDonald (2009), and a more detailed one in Frühwirth-Schnatter (2006).

Agglomerative hierarchical clustering (*hc*)

hc starts each data as a cluster of its own, and successively merges the closest pair of clusters to form a new, larger cluster until the desired cluster structure is obtained. The result of the *hc* is a dendrogram, representing the nested grouping of data and similarity levels at which grouping changes. The clustering of data is obtained by cutting empirically the dendrogram at the desired similarity level.

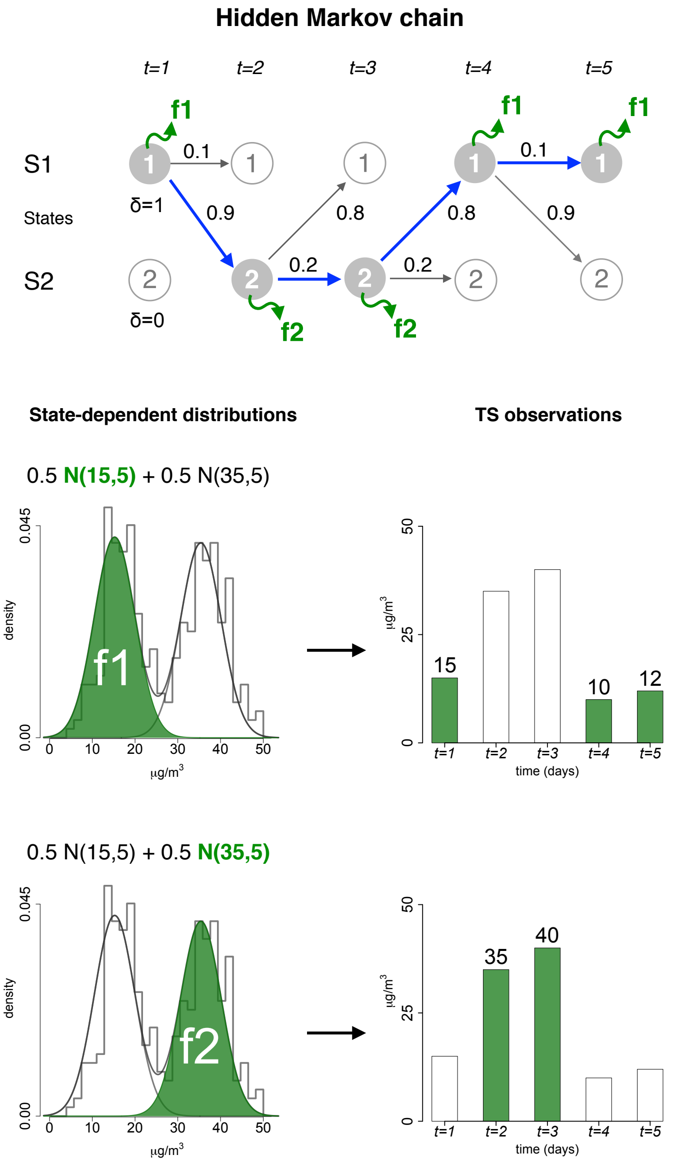

$$\mathbf{A} = \begin{array}{c} \mathcal{S} \\ \text{from 1} \\ \text{from 2} \end{array} \begin{array}{c} \begin{array}{cc} \text{to 1} & \text{to 2} \end{array} \\ \left(\begin{array}{cc} 0.1 & 0.9 \\ 0.8 & 0.2 \end{array} \right) \end{array}$$

Figure SM.1. Example of a time series modeling with a 2-states (k=2) *hmm*. **A** represents the transition probability matrix of the unobserved Markov chain.

SM.2

Tables SM.1-SM.5. Characterization of background pollution in studied sites, from 2005 to 2015, using hidden Markov models (in μg/m^3): mean (m), standard deviation (sd), representativeness along the year (%) of the background pollution (first cluster). Annual mean (M) and standard deviation (SD) values from the monitored pollutant TS. In each of the TS, T indicates the length (days) and k the number of cluster detected.

City	Site	Pollutant	Year	T	k	%	m	sd	M	SD
Córdoba	Asomadilla	CO	2005	178	3	38	295.3	45.8	406.1	120.6
			2006	312	4	56	333.6	48.0	498.4	219.0
			2007	164	5	10	153.0	38.8	540.1	253.9
			2010	62	2	63	128.1	32.6	162.0	59.4
			2011	363	4	14	74.6	16.5	266.6	144.0
			2012	311	4	57	177.3	48.4	305.4	240.1
			2013	364	4	16	99.3	20.2	191.3	77.5
			2014	319	3	32	161.9	36.9	256.1	92.0
			2015	55	1	100	452.7	55.9	452.8	56.4
		NO_2	2005	160	2	19	14.8	6.5	22.6	8.1
			2006	295	2	45	19.6	5.8	26.9	9.7
			2007	353	3	45	17.6	3.4	25.0	8.4
			2008	349	2	66	18.3	5.2	21.8	8.9
			2009	345	3	41	12.5	3.5	18.8	7.5
			2010	323	2	63	13.2	3.7	16.7	6.2
			2011	359	2	67	13.1	4.3	16.2	6.4
			2012	343	3	35	9.5	2.7	15.8	6.5
			2013	312	3	24	8.8	1.7	15.8	5.5
			2014	276	2	55	9.7	3.4	14.1	6.3
			2015	295	2	52	12.5	3.7	16.9	6.9
		O_3	2005	182	3	46	39.5	11.5	61.4	23.5
			2006	339	3	34	31.6	10.9	58.6	24.3
			2007	359	4	19	23.7	6.5	56.7	22.7
			2008	357	4	21	27.7	10.5	57.9	23.4
			2009	353	4	21	29.9	9.2	63.0	22.5
			2010	338	4	24	36.0	11.8	65.0	21.6
			2011	355	4	16	25.6	8.6	60.4	22.0
			2012	334	3	21	28.6	7.6	62.3	22.4
			2013	362	3	37	42.8	12.2	65.7	22.4
			2014	331	4	17	29.1	8.8	62.5	23.0
			2015	365	4	19	33.2	6.2	64.6	24.4
		PM_{10}	2005	161	2	49	17.4	5.8	25.1	11.4
			2006	339	3	35	17.6	10.2	36.3	20.8
			2007	359	4	38	23.1	10.0	37.2	20.8
			2008	350	4	24	14.4	8.6	26.2	12.8
			2009	353	3	36	17.3	10.2	30.6	13.2
			2010	342	4	40	14.8	10.0	26.5	14.9
			2011	343	3	14	12.1	8.6	24.4	9.8
			2012	251	3	66	18.5	10.2	25.5	14.7
			2013	361	4	29	10.8	10.0	18.9	7.8
			2014	326	3	63	14.0	8.6	19.2	10.1
			2015	363	4	34	13.3	10.2	21.9	9.6

City	Site	Pollutant	Year	T	k	%	m	sd	M	SD
Jaén	Bailén	CO	2007	158	4	18	360.1	100.4	638.4	242.3
			2008	343	5	13	250.8	41.2	569.1	198.0
			2009	338	4	9	269.2	37.7	574.3	194.5
			2010	331	4	19	236.3	65.5	493.1	190.9
			2011	339	4	31	84.1	8.1	204.3	207.8
			2012	361	5	16	95.7	2.0	158.7	77.7
			2013	361	5	10	82.5	1.7	144.5	82.2
			2014	362	4	15	90.7	14.8	157.3	78.5
			2015	354	4	31	61.4	6.7	124.2	106.6
		NO_2	2005	353	3	24	16.7	3.8	29.9	11.5
			2006	317	3	40	23.3	6.4	33.3	11.6
			2007	274	3	26	20.0	5.2	33.1	12.2
			2008	332	3	30	16.1	4.1	26.9	10.3
			2009	340	3	10	7.4	1.6	23.7	11.4
			2010	316	3	35	13.9	3.7	23.0	9.3
			2011	344	3	31	11.8	3.6	22.6	10.0
			2012	361	3	27	8.5	2.7	20.8	10.4
			2013	362	3	15	6.3	2.1	18.8	9.4
			2014	362	4	22	7.6	1.6	18.3	9.5
			2015	354	3	33	10.6	3.8	21.4	10.8
		O_3	2010	195	3	38	36.9	14.2	59.7	22.8
			2011	344	3	27	25.5	8.4	53.2	20.6
			2012	361	3	20	25.6	8.4	58.1	22.5
			2013	357	4	34	34.1	9.9	57.7	20.9
			2014	361	4	15	23.4	7.4	58.1	23.2
			2015	339	4	39	32.6	11.2	58.0	24.6
		PM_{10}	2005	310	3	25	37.1	11.3	65.3	28.1
			2006	356	3	19	29.9	7.1	62.3	26.2
			2007	310	3	51	35.7	10.7	50.8	26.8
			2008	346	3	34	21.9	6.8	39.4	19.7
			2009	336	3	40	19.3	5.9	34.5	16.1
			2010	322	4	29	19.6	5.1	39.9	21.2
			2011	339	4	20	23.0	6.1	40.7	16.8
			2012	355	4	21	18.3	4.4	35.7	19.1
			2013	355	4	20	14.8	3.6	30.4	12.4
			2014	360	3	47	21.2	6.4	33.4	16.8
			2015	351	3	42	23.3	6.8	37.9	19.2

City	Site	Pollutant	Year	T	k	%	m	sd	M	SD
			2005	361	3	30	13.3	3.4	25.9	11.9
			2006	200	2	51	17.3	6.5	27.2	12.8
			2007	360	3	33	13.6	3.7	24.6	11.7
			2008	366	3	44	13.8	4.0	21.9	9.8
			2009	358	3	43	11.9	3.2	20.0	10.1
		NO_2	2010	362	3	39	8.4	2.5	17.5	10.4
			2011	365	3	59	11.5	4.6	17.8	10.0
			2012	365	3	23	7.4	1.5	17.3	9.1
			2013	344	3	16	5.6	1.3	14.9	8.1
			2014	340	3	45	7.1	1.9	13.3	8.3
			2015	275	3	39	9.1	3.1	17.9	10.2
			2005	361	4	13	31.8	7.2	63.2	22.0
			2006	357	3	30	34.5	8.9	60.9	22.5
			2007	361	4	17	29.6	6.8	60.0	20.1
			2008	366	4	42	41.0	11.5	62.8	23.4
			2009	361	3	17	34.9	10.7	66.9	21.9
Seville	Aljarafe	O_3	2010	365	4	33	42.8	14.8	64.4	21.1
			2011	361	3	15	26.7	5.7	61.6	21.9
			2012	365	3	26	35.5	9.1	64.4	21.9
			2013	364	3	35	43.5	10.3	66.8	21.3
			2014	365	3	15	29.7	9.0	60.3	19.8
			2015	359	4	12	30.9	8.6	62.8	18.7
			2005	151	2	65	35.0	11.0	43.6	16.9
			2006	169	3	34	33.0	7.2	52.7	20.0
			2007	253	3	57	34.0	8.5	45.6	23.2
			2008	364	3	44	22.8	6.1	36.1	20.0
			2009	354	3	27	19.0	5.0	32.9	12.8
		PM_{10}	2010	361	4	37	19.2	4.3	33.3	16.6
			2011	365	3	24	18.2	4.7	35.6	16.2
			2012	362	3	49	19.9	5.3	30.2	14.9
			2013	351	3	40	17.1	3.7	26.0	9.4
			2014	354	4	32	14.5	3.4	26.6	13.3
			2015	350	4	29	15.6	3.6	29.7	13.4

City	Site	Pollutant	Year	T	k	%	m	sd	M	SD
Seville	Bermejales	CO	2005	346	4	31	87.4	43.8	340.2	293.2
			2006	357	5	12	103.4	44.5	338.9	196.2
			2007	322	4	2	30.0	7.9	421.2	204.7
			2008	329	4	13	215.6	36.2	531.4	190.5
			2009	363	4	29	465.5	44.8	629.8	164.3
			2010	353	5	11	275.5	89.5	549.8	146.9
			2011	365	4	28	446.8	66.0	654.6	166.3
			2012	365	5	15	269.9	43.2	458.2	146.0
			2013	339	4	18	185.7	82.8	459.0	184.7
			2014	313	5	15	316.5	31.0	381.2	109.4
			2015	338	5	4	323.0	63.2	662.4	168.6
		NO_2	2005	329	3	43	20.8	7.7	31.9	12.9
			2006	360	3	59	18.7	7.6	27.3	13.7
			2007	327	3	44	15.1	7.4	29.0	16.8
			2008	337	3	44	23.9	7.2	33.4	14.1
			2009	335	2	52	28.0	8.4	37.5	13.7
			2010	339	3	68	23.1	8.8	32.9	18.8
			2011	364	3	20	11.7	6.1	28.1	15.0
			2012	342	3	40	9.2	5.8	21.6	14.2
			2013	330	3	35	9.6	4.8	21.5	12.8
			2014	320	2	72	20.4	6.4	26.5	10.6
			2015	275	3	58	18.7	5.8	27.4	13.2
		O_3	2005	355	3	37	25.5	10.2	46.4	20.7
			2006	362	3	27	21.9	6.9	48.1	21.6
			2007	330	3	20	18.0	5.3	47.3	22.1
			2008	356	3	26	21.6	8.6	48.3	22.1
			2009	358	3	22	21.9	8.2	49.1	20.8
			2010	349	3	29	30.1	11.5	54.0	20.5
			2011	365	4	16	17.9	5.8	50.9	21.4
			2012	365	3	19	23.3	6.5	51.5	19.5
			2013	359	3	40	34.6	11.9	56.6	22.2
			2014	344	3	23	23.7	9.3	55.1	22.1
			2015	362	4	20	29.5	8.3	54.2	18.9
		PM_{10}	2005	122	3	19	15.8	5.7	28.2	10.7
			2006	337	3	39	15.2	3.4	24.2	10.1
			2007	311	3	39	16.4	4.2	23.5	9.7
			2008	355	4	41	17.8	5.4	37.2	27.9
			2009	346	3	41	13.3	2.7	17.9	10.0
			2010	365	3	52	13.9	3.2	18.9	8.5
			2011	45	1	100	43.9	17.2	43.9	17.4
			2012	341	3	28	18.1	5.4	32.8	15.6
			2013	335	3	39	17.0	5.1	28.1	11.9
			2014	340	3	49	15.2	4.6	23.8	10.9
			2015	342	3	31	21.0	5.7	35.1	14.7

City	Site	Pollutant	Year	T	k	%	m	sd	M	SD
Seville	Torneo	CO	2005	259	3	20	352.0	101.3	767.8	309.1
			2006	345	4	26	210.3	82.1	471.3	262.0
			2007	344	4	39	253.2	89.5	612.8	357.0
			2008	302	4	31	254.0	82.7	608.6	342.1
			2009	357	4	12	187.1	129.9	695.8	281.2
			2010	350	4	17	467.1	82.4	611.7	108.3
			2011	365	5	11	361.5	39.5	610.8	169.5
			2012	365	3	41	303.0	72.8	471.3	193.7
			2013	327	4	6	153.0	51.4	421.0	173.3
			2014	349	3	29	328.8	70.9	473.8	143.4
			2015	351	3	52	200.6	53.0	291.3	122.6
		NO_2	2005	341	3	37	35.9	8.4	49.9	14.5
			2006	363	2	48	41.0	9.1	49.7	12.2
			2007	356	3	28	35.2	7.1	48.2	11.5
			2008	334	3	31	25.9	8.4	39.4	12.9
			2009	356	2	56	23.3	7.8	30.7	11.9
			2010	342	4	31	16.9	7.0	37.5	21.5
			2011	359	2	55	37.5	8.2	45.6	12.2
			2012	365	3	22	13.4	6.8	33.7	15.4
			2013	318	3	33	24.7	7.2	36.7	11.6
			2014	360	2	61	31.7	6.3	37.4	9.2
			2015	360	3	25	27.4	4.3	39.8	10.8
		O_3	2005	358	4	12	16.5	4.9	42.9	18.8
			2006	363	3	28	24.9	4.6	46.2	17.8
			2007	352	3	23	25.0	4.8	46.1	16.5
			2008	334	3	21	23.2	5.4	43.2	16.0
			2009	352	4	19	22.6	6.4	45.9	16.7
			2010	350	3	35	28.1	10.3	45.6	17.0
			2011	365	4	23	17.6	6.8	40.9	17.6
			2012	363	3	19	14.7	4.3	39.2	17.7
			2013	330	3	35	23.5	8.4	44.1	18.7
			2014	360	4	15	14.9	4.4	40.2	17.4
			2015	365	4	19	17.0	4.8	41.1	17.9
		PM_{10}	2005	309	2	53	26.0	6.9	33.1	10.9
			2006	98	2	60	16.2	4.6	22.6	9.8
			2007	274	3	22	22.1	4.4	31.5	9.3
			2008	294	2	81	24.7	3.4	26.1	5.7
			2009	332	2	71	22.2	3.2	24.6	5.3
			2010	342	3	34	19.3	2.5	27.4	12.1
			2011	333	3	25	32.4	6.5	42.6	11.4
			2012	312	3	40	19.6	5.2	29.7	12.2
			2013	350	3	42	20.8	5.0	30.2	10.4
			2014	358	4	47	19.0	4.5	28.4	13.0
			2015	355	3	35	26.7	6.3	37.9	11.7

SM.3

The next are the graphical representation of the background pollution evolution in Aljarafe (Seville), Asomadilla (Córdoba), Bailén (Jaén) and Bermejales (Seville) sites, from 2005 to 2015.

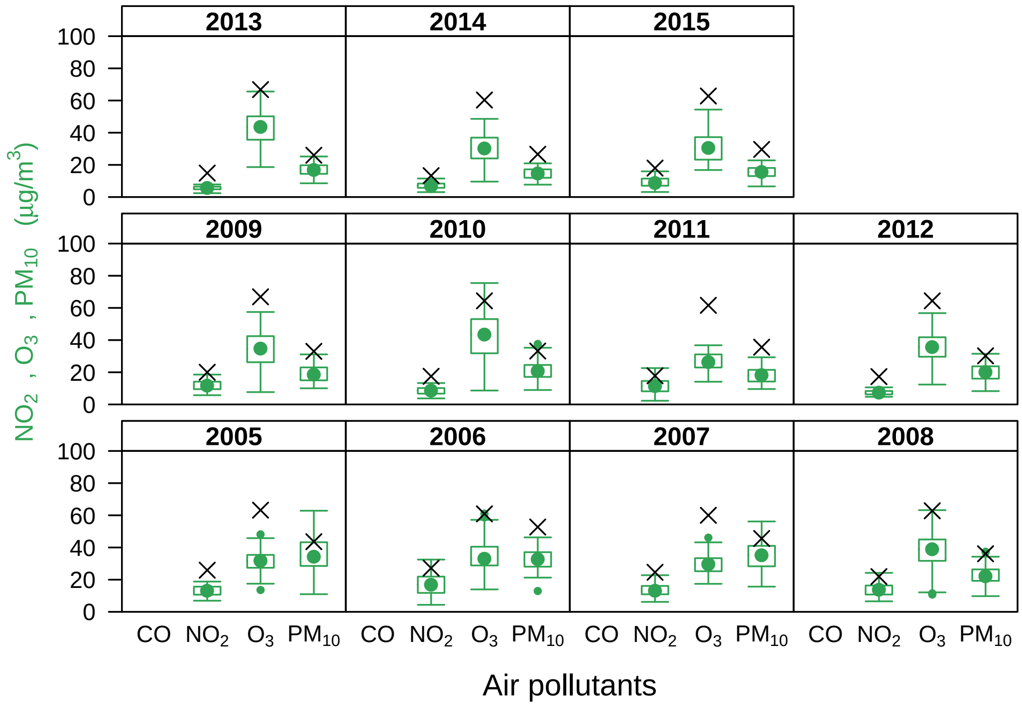

Figure SM.2. Aljarafe site.

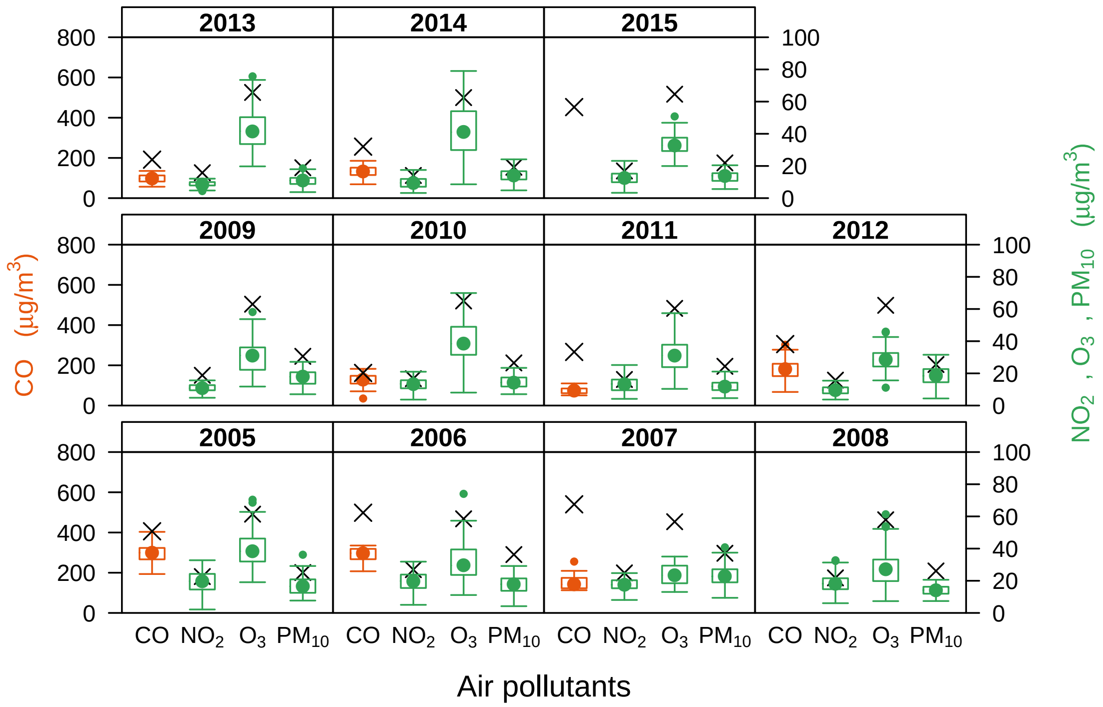

Figure SM.3. Asomadilla site.

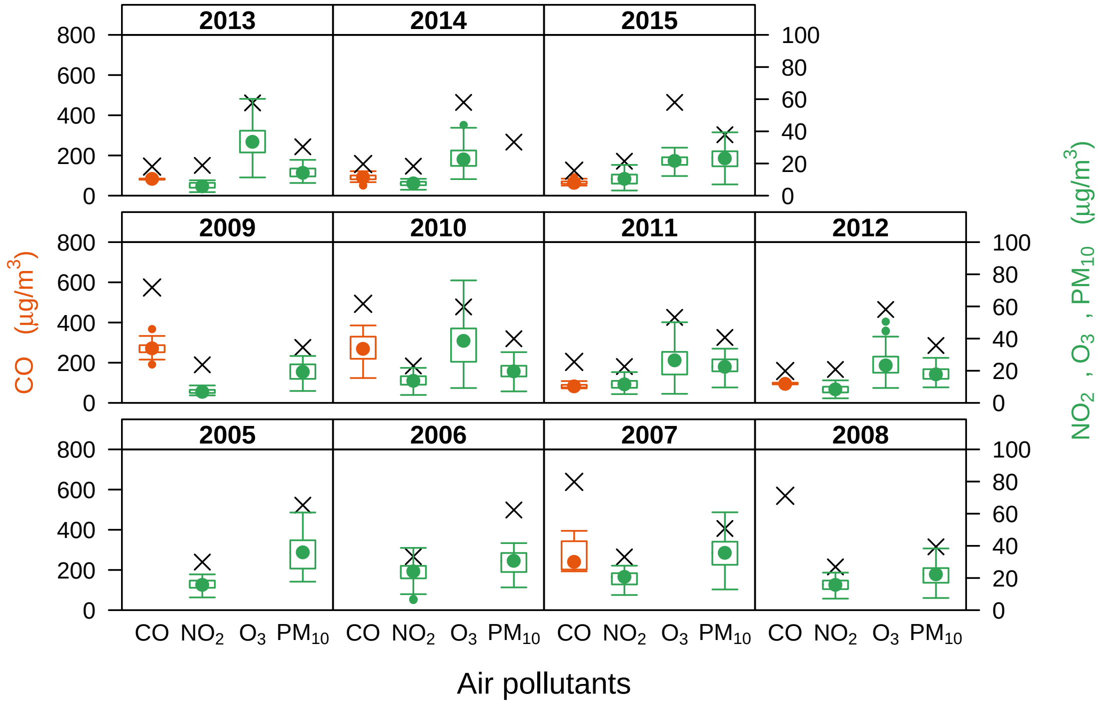

Figure SM.4. Bailén site.

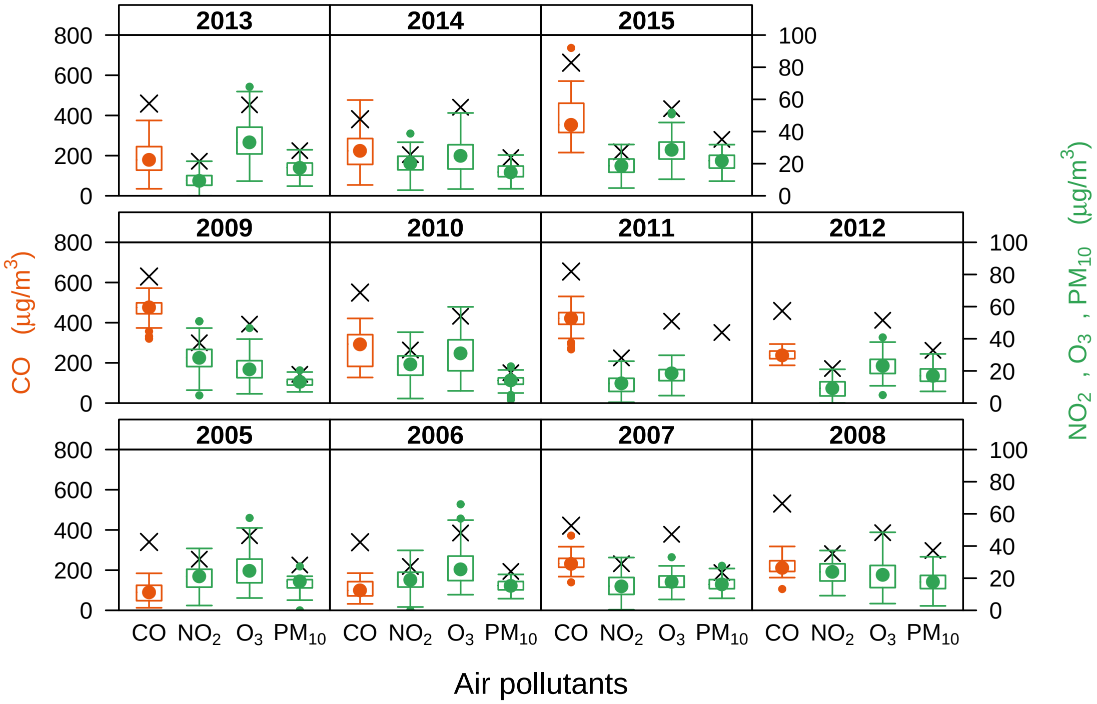

Figure SM.5. Bermejales site.

Supplementary Material references

Frühwirth-Schnatter, S. Finite mixture and Markov switching models. Springer. 2006. p. 493.

Hartigan, J.A., Wong, M.A. A K-means clustering algorithm. Journal of the Royal Statistical Society. Series C (Applied Statistics) 1979, 28(1): 100-108.

Ibe, OC. Hidden Markov Models. In: Markov process for Stochastic Modeling, 2nd ed. Elsevier, 2013. p. 417-451. http://dx.doi.org/10.1016/B978-0-12-407795-9.00014-1

Raykov, Y.P., Boukouvalas, A., Baig, F., Little, M.A. What to do when $k - means$ clustering fails: a simple yet principled alternative algorithm. Plos One 2016, 11(9): 1-28.

Venables, WN, Smith, DM, R Core Team. An introduction to R. Notes on R: A programming environment for data analysis and graphics. 2009. Available from: http://cran.r-project.org/doc/manuals/R-intro.pdf.

Visser I, Raijmakers MEJ, Van der Maas HLJ. Hidden Markov Models for Individual Time Series. In: Valsiner, J, Molenaar, PCM, Lyra MCDP, Chaudhary, N, editors. Dynamic Process Methodology in the Social and Developmental Sciences. Springer-Verlag, New York, 2009. p. 269-289.

Visser, I., Speekenbrink, M. depmixS4: An R package for Hidden Markov Models. Journal of Statistical Software; 2010, 36(7):1-21. Available from:
http://cran.r-project.org/web/packages/depmixS4/vignettes/depmixS4.pdf

Visser, I. Seven things to remember about hidden Markov models: A tutorial on Markovian models for time series. Journal of Mathematical Psychology 2011; 55: 403-415. http://doi.org/10.1016/j.jmp.2011.08.002

Zucchini, W., MacDonald, I. Hidden Markov Models for Time Series: An Introduction Using R. Monographs on Statistics and Applied Probability. CRC Press, Boca Raton. 2009.
